# 

**Published:** 1886-10

**Authors:** 


### Ohio State Dental Society.

The second annual meeting of the Ohio State Dental Society (reorganized) will be held in Toledo, beginning Tuesday, October 26th, at ten o'clock A.M.

Preparations are being made for an interesting meeting, and it is to be hoped that there will be present a number of the profession of this and adjoining States. Especially is it desired that every member of the Society be present.

The Society is starting on a new career and with every promise of renewed vigor and better results than ever before.

Ohio was one of the first to have a State society, and an efficient law regulating the practice of dentistry, and the profession should certainly have a pride in sustaining the organization. The following program of subjects for discussion has been prepared by the Executive Committee:

1. What Should be the Treatment in Cases of Impacted Third Molars; paper by C. R. Butler, M. D.
2. Method of Investigating the Decay of the Teeth.
3. Obtundents for Sensitive Dentine—Their Value and Mode of Action.
4. Materia Medica and New Remedies.
5. Filling Teeth by the Herbst Method; demonstrated by Dr. Rehwinkel.
6. New Methods of Supplying Artificial Teeth; demonstrated by C. H. Land and others.
7. Correction of Oral Deformities; demonstrated by Dr. Geo. W. Keely.

Exhibition of new instruments and appliances at such time as may be determined by the Society.

The meeting will be held in the Council Chamber in Masonic Building; and with Dr. F. H. Rehwinkel for President, Dr. J. R. Callahan for Secretary, Dr. Geo. W. Keely as Treasurer, and Dr. C. H. Harroun, Chairman of the Committee of Arrangements, no one can doubt but the meeting will be a grand success.

So come one and all, and bring whatever you can to add to the interest and the profit of the occasion.

### CONTENTS.

### COMMUNICATIONS.

A MONTHLY MAGAZINE DEVOTED TO THE INTERESTS OF THE

**DENTAL PROFESSION.**

*Terms Reduced to* $2.00 *Per Year. Single Copies,* 25 *Cents.*

**EDITED BY PROF. J. TAFT, D.D.S.**

—AND—

**Published by SAM'L A. CROCKER & CO., Ohio Dental and Surgical Depot.**

The volume commences in January and closes in December.

The REGISTER being issued monthly is always fresh, therefore Indispensable to the practitioner who desires to keep posted up with the new inventions and improvements which are daily developed. Neither expense nor effort will be spared to give value and variety to its contents. Articles will be illustrated with well executed engravings whenever they will add to the interest or assist to explanation.

Its extensive circulation and frequency of issue make it one of the BEST DENTAL ADVERTISING MEDIUMS in the United States.

All subscriptions must begin with January or July.

Local or special notices, five lines or less, will be inserted for $3 each insertion; over five lines, 50 cts. per line.

All advertising bills payable in advance, or at furthest, after the issue of the first number containing the advertisement. All transient advertisements to be paid for in advance.

☞CONTRIBUTIONS SOLICITED.

Exclusively Editorial Communications should be addressed to the Editor.

The latest number of the REGISTER may be ordered with or without other goods at any time, and all letters should be addressed to

**SAM'L A. CROCKER & CO., Ohio Dental and Surgical Depot, Cincinnati, O.**

